# Immuno-informatics analyses of important esophageal cancer associated viruses for multi-epitope vaccine design

**DOI:** 10.3389/fimmu.2025.1587224

**Published:** 2025-07-08

**Authors:** Zafer Saad Al Shehri

**Affiliations:** Department of Medical Laboratories, College of Applied Medical Sciences, Shaqra University, Dawadmi, Saudi Arabia

**Keywords:** esophageal cancer, Epstein-Barr virus (EBV), human papillomavirus (HPV), human cytomegalovirus (hcmv), molecular docking simulation, multi-epitope vaccine

## Abstract

**Introduction:**

Esophageal cancer (EC) is a highly lethal malignancy characterized by the uncontrolled proliferation of cancerous cells within the esophagus. Despite recent advancements in therapeutic strategies, the prognosis remains poor, underscoring the urgent need for novel preventive and therapeutic approaches. Notably, several oncogenic viruses have been implicated in EC pathogenesis, prompting the exploration of epitope-based vaccines through immunoinformatics.

**Methods:**

Using immunoinformatics and bioinformatics approaches, we designed a novel multi-epitope vaccine targeting viral agents associated with EC. Protein sequences of ten viral candidates were retrieved from the UniProt database and evaluated for antigenicity using the VaxiJen server. Five highly antigenic proteins derived from Human Cytomegalovirus (HCMV), Human Papillomavirus (HPV), Human Herpesvirus 8 (HHV-8), Human Immunodeficiency Virus (HIV), and Epstein–Barr Virus (EBV) were selected. T cell (CTL and HTL) and B cell (LBL) epitopes were predicted and screened for immunogenicity, allergenicity, and toxicity. The final vaccine construct incorporated β-defensin as an adjuvant and included 3 HTL, 8 CTL, and 8 LBL epitopes. Molecular docking and molecular dynamics (MD) simulations were conducted to assess the binding affinity of the vaccine with Toll-like receptor 3 (TLR3). In silico cloning was also performed using the pET-28a(+) vector in *Escherichia coli* strain K12.

**Results:**

The designed vaccine was found to be antigenic, non-allergenic, and non-toxic. Molecular docking revealed strong binding affinity between the vaccine construct and TLR3, which was further supported by MD simulation results indicating stable complex formation. Codon optimization and in silico cloning confirmed the high expression potential of the vaccine in the *E. coli* expression system.

**Discussion:**

The in silico analyses suggest that the developed multi-epitope vaccine construct is a promising candidate for preventing EC associated with viral infections. While these findings are encouraging, further experimental validation through in vitro and in vivo studies is essential to confirm the vaccine's safety, immunogenicity, and protective efficacy.

## 1 Introduction

Esophageal cancer (EC) is a cancer that begins in the esophagus, the tube that connects the throat and stomach. It typically manifests as squamous cell carcinoma or adenocarcinoma (1, 2). Mortality rates and age-standardized incidence (ASMR and ASIR) for EC were 5.0 and 4.3 per 100,000, respectively, with an estimated 511,054 new cases and 445,391 related deaths reported worldwide in 2022. East Asia and East Africa were found to have the highest rates. China alone was responsible for more than 40% of cases and fatalities worldwide. Male rates were consistently higher, and the burden was greatest in countries with high Human Development Indexes (HDIs). Due to age and population expansion, it is predicted that cases and deaths worldwide will increase by more than 80% by 2050 (3). Symptoms of EC include unintentional weight loss, difficulty swallowing (dysphagia), chest pain or discomfort, hoarseness, persistent cough, and, in some cases, regurgitation of food or vomiting. As the disease progresses, these symptoms may become more severe and debilitating. Barrett’s esophagus, excessive alcohol consumption, chronic gastroesophageal reflux disease (GERD), obesity, smoking, and a low-vegetable and fruit diet are primary risk factors for EC (4–8).

Because of its aggressive nature, rapid progression, and often delayed diagnosis, EC is particularly deadly, with a survival rate of five years less than 20% in many cases. Recurrence and metastasis continue to be major obstacles despite of the availability of treatments such as radiation, chemotherapy, and surgery (9–11). A multi-epitope strategy is required because it can improve immunotherapy efficacy, boost the immune response by targeting multiple tumor-associated antigens, and possibly lower the risk of cancer cells evading the immune system (12).

Human Papillomavirus (HPV), Human Herpesvirus 8 (HHV-8), Human Cytomegalovirus (HCMV), Epstein-Barr Virus (EBV), and Human Immunodeficiency Virus (HIV) can all be considered causative agents of EC due to their ability to cause chronic inflammation, promote cellular transformation, and evade immune detection. The E7 and E6 oncoproteins of HPV cause the cell cycle to become unbalanced, which can result in cancerous development (13, 14). The Latent Membrane Protein 1 (LMP1) proteins & Epstein-Barr Nuclear Antigen 1 (EBNA1) of EBV impede apoptosis and foster a pro-tumorigenic environment (15, 16). HCMV’s Immediate Early (IE) proteins and UL97 kinase promote cell proliferation and viability (17). The viral interleukin-6 (vIL-6) and Latency-associated nuclear antigen (LANA)proteins of HHV-8 promote cell proliferation and angiogenesis. HIV-induced immunosuppression promotes the persistence of these oncogenic viruses, increasing the risk of cancer development (18, 19). These viruses, combined, contribute to the complex pathogenesis of esophageal cancer through their oncogenic properties and interactions with host cellular mechanisms. Despite the availability of antivirals and vaccines (e.g., HPV), obstacles such as latent infection, immune evasion, and a lack of targeted therapeutics for particular viruses impede effective prevention and therapy, emphasizing the need for multi-targeted therapeutic techniques such as pan-viral cancer vaccines.

The multi-epitope vaccine designed in this study is intended as a therapeutic intervention targeting viral antigens associated with esophageal cancer. Unlike preventive vaccines that aim to block infection, therapeutic vaccines seek to stimulate the immune system to recognize and eliminate cancer cells harboring viral proteins. This approach leverages the unique viral signatures present in tumor cells to enhance anti-tumor immunity and represents a promising strategy in cancer immunotherapy.

## 2 Materials and methods

### 2.1 Retrieval of viral sequences associated with EC

The ten viral proteins: E6 and E7 oncoproteins (HPV); LMP1 and EBNA1 (EBV); Immediate Early (IE) protein and UL97 kinase (HCMV); LANA and vIL-6 (HHV-8); and Tat and Nef (HIV) were chosen for the design of a multi-epitope vaccine to HPV, EBV, HCMV, HHV-8, HIV. To test their appropriateness as vaccine candidates, the antigenic potential of these proteins was determined with the VaxiJen server (20).

### 2.2 Epitopes prediction and selection phase

Predictions made during the epitope selection phase included B-cell epitopes as well as both helper T lymphocyte (HTL) and cytotoxic T lymphocyte (CTL) epitopes. B-cell epitopes are of paramount importance to the creation of peptide vaccines, diagnostics for diseases, and research on allergy (21). ABCPred predicted linear B cell epitopes (LBL) (22).

The Immune Epitope Database (IEDB) server analyzed all targeted proteins for MHC class I alleles, a critical step in cytotoxic T lymphocyte (CTL) screening to confirm peptide binding to major histocompatibility complex class I molecules (23). Depending on the MHC allele, length preferences can change, but we considered 9-mer epitopes.

The detection of epitope candidates in cancer, infectious agents, allergies, and autoantigens is done directly using HLA class II molecules (23). In this study, 15-mer peptides were chosen, a consensus method was used, and they were compared to 27 HLA alleles to determine HTL epitopes using the IEDB server. In human antigen-presenting cells, high binding power epitopes (MHC-I and MHC-II) were found using adjusted rank < 2 filtering (23).

VaxiJen version 2.0, Allergen version FP1.0, ToxinPred, and the MHCI immunogenicity servers were used to further assess the predicted epitopes (20, 21, 24, 25). Ultimately, HTL epitopes that trigger IFN-γ were sought using the IFNepitope online server (26).

### 2.3 Epitopes population coverage

T cells identify a combination of a pathogen-derived epitope and a particular MHC molecule. Different ethnic groups express certain HLA alleles at very different frequencies. T-cell epitope-based vaccines for PC were designed and developed using the IEDB population coverage server (27, 28). Population coverage for T cell (MHC I & MHC II) epitopes across various ethnic groups was examined in this study.

### 2.4 Vaccine construction

To create an effective multi-epitope vaccination, CTL, HTL, and LBL were combined with the right linker. Three distinct linkers—AAY, GPGPG, and KK—bound the CTL, HTL, and LBL epitopes. Because they are necessary for generating functional region separation, a broad conformation (flexibility), and protein folding—all of which enhance the stability of the protein structure—these linkers are used. According to earlier studies, the linkers were chosen for their length, stiffness, flexibility, and efficacy (29, 30). Most of the time, a vaccine’s epitopes by themselves are insufficient to trigger an immune response (28). The adaptive and innate immune systems must be stimulated by carriers abundant in immunostimulatory adjuvants. The EAAAK linker was used to join the β-defensin adjuvant to the N-terminal of the vaccine construct. β-defensin was chosen for its capacity to boost antigen absorption and activate dendritic and T cells, resulting in powerful adaptive immunological responses. Research indicates that β-defensin-antigen fusions enhance tumor-specific immunity in cancer vaccines, making them a promising adjuvant (31–33).

### 2.5 Post analysis of vaccine

Physical and chemical properties are studied to determine a protein’s structural and functional properties. The final vaccine construct’s chemical and physical properties were evaluated using the ProtParam server (34). We comprehend the activity, stability, and nature of proteins by utilizing the diverse physical and chemical parameters available on this server, including the amino acid composition, extinction coefficient, aliphatic index, theoretical pI, instability index, atomic composition, and molecular weight. Protein solubility is another important factor to consider when designing vaccines, as it is important for therapeutic and industrial applications. Protein solubility was predicted by employing the SOLpro server (35). The potential of the vaccine to elicit allergic reactions can be predicted through allergenicity testing. Thus, AllerTop was utilized (34). Structural antigenicity was examined using VaxiJen v2.0 (36). Predicting a protein’s secondary structure is a challenging task in bioinformatics. Local conformation proteins have three distinct secondary structures: β-strand, α-helix, and coil region. The secondary vaccine structure was analyzed using the SOPMA server (37). 3Dpro server was used to predict the vaccine’s three-dimensional structure (38). The community focused on structure prediction emphasizes enhancing template-based structure models, going beyond the present level of accuracy in template information. Therefore, the vaccines ‘s structure was improved using the GalaxyRefine server (39). ProSA-web servers and UCLA-DOE LAB were utilized to assess the reliability and caliber of the chosen three-dimensional structure (40–42). The Ramachandran diagram calculates the residues and probability distribution of dihedral angles ψ and φ in the backbone, illustrating the structure’s quality by determining the amount and percentage of residues. The Ellipro server predicted vaccine conformational/linear B cell epitopes, with the vaccine’s 3D structure as an input (43).

### 2.6 Disulfide engineering

Disulfide bridge formation between cysteine residues plays a pivotal role in maintaining the structural integrity and functional conformation of proteins and peptides. In this study, the Disulfide by Design version 2.0 (DbD2) server was employed to strategically engineer disulfide bonds within the vaccine construct, with the aim of enhancing molecular stability by elevating the free energy of the unfolded state and reducing conformational entropy, thereby contributing to the refinement of the vaccine’s three-dimensional architecture (44).

### 2.7 Immune simulation

The C-IMMSIM server is a bioinformatics-based immunological response simulator that predicts B and T cell epitopes (45). C-ImmSim describes the humoral and cellular profiles of a mammal immune system’s response to a vaccine using the Celada-Seiden model. The study uses pictures from the myeloid and lymphoid lineages, including dendritic cells and macrophages. The simulated parameters include three vaccination doses, a vaccine without lipopolysaccharides, and adjustments to the simulation’s volume and steps. The goal is to produce an effective and durable immune response. There is no change to the other parameter, “Random Seed”. The immune response modeling can be completed in about 350 days (1050 × 8 h)/(24 h)) since one simulation step is equal to eight hours (8 h) of real-time.

### 2.8 Molecular docking

Docking analysis was used to determine how effectively vaccine constructs bound to the TLR3 immune cell receptor. TLR3 was chosen for molecular docking because of its capacity to identify viral double-stranded RNA and initiate potent type I interferon and cytotoxic T cell responses—both of which are essential for antitumor immunity. Activation of TLR3 stimulates apoptosis and immunological activation without causing severe inflammation, in contrast to TLR4 or TLR9. TLR3 expression has also been associated with improved prognosis and immune infiltration in esophageal cancer, which supports its applicability as a docking target (46–48). The HADDOCK-v-2.4 server was utilized for protein-protein interactions, with interactions assessed using PDBsum and complexes visualized using PyMOL version 1.3 (49–51).

### 2.9 MD simulation

MD simulations were run with the Desmond software of Schrodinger suite at 100 ns (52). Molecular docking-created protein-protein complexes were analyzed using MDS to investigate their dynamic interactions. Before optimization and minimization, the complex underwent preprocessing. During the energy minimization process, the OPLS_2005 force field was integrated with the Transferable Intermolecular Potential with 3 Points (TIP3P) water model and an orthorhombic simulation box to accurately simulate the solvent environment (53, 54). To achieve system neutrality, counterions were introduced, and physiological conditions were replicated by incorporating 0.15 M NaCl. Before initiating the simulation, the complex underwent relaxation, followed by execution under an NPT ensemble maintained at 300 K and 1 atm pressure. Simulation trajectories were recorded at 50 ps intervals for subsequent analysis.

### 2.10 *In silico* cloning

**Figure 1:**
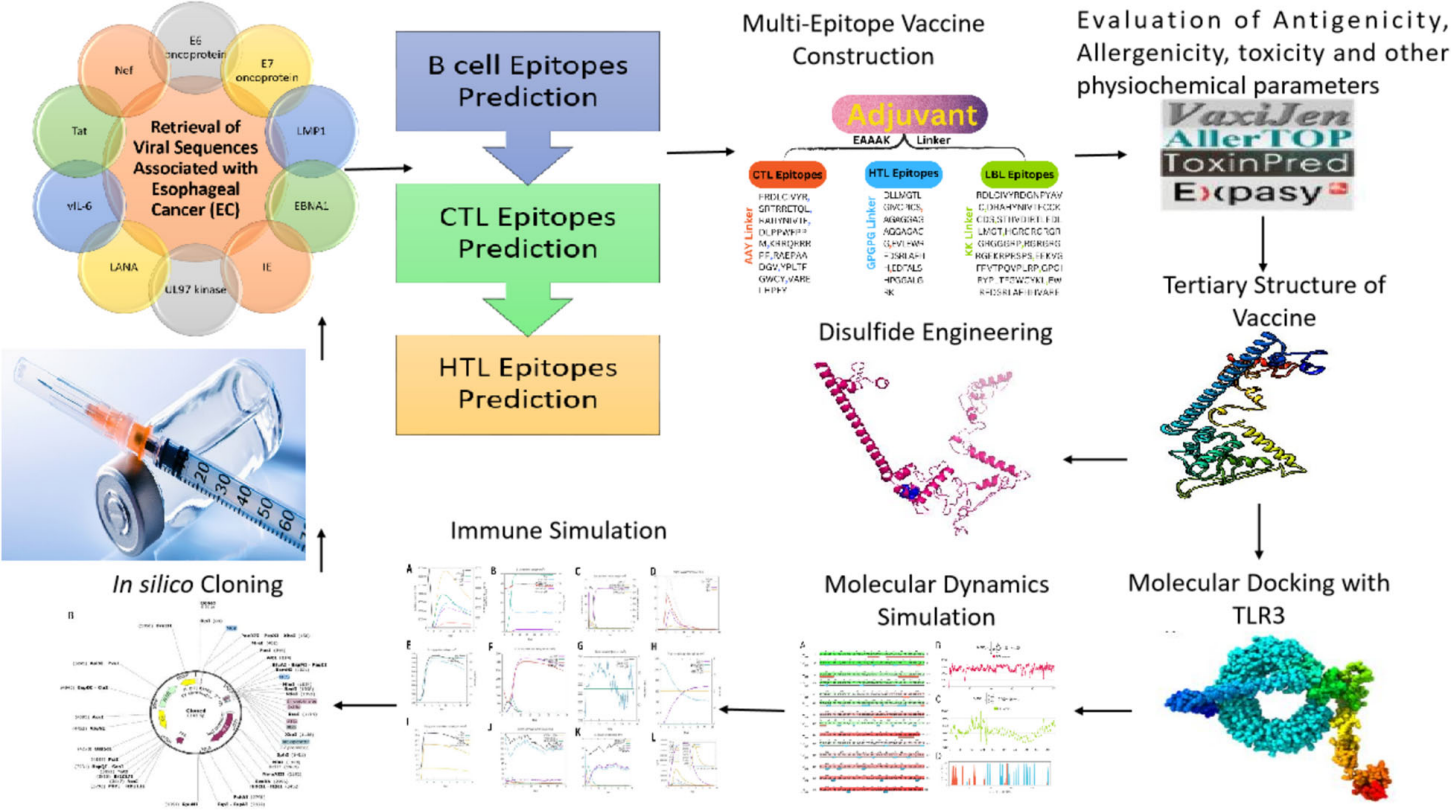
Graphical abstract.

Nowadays, most sequenced prokaryotes require a group of prediction servers to change the target gene codon’s mode of action. Eukaryotic gene expression hosts are chosen to enhance the synthesis of heterologous proteins. *E. Coli* strain K12 and the Java Codon Adaptation Tool (JCat) server was used to quantify the expression level of a multi-epitope vaccine (55). For every query sequence, JCat determines the GC content and CAI value, identifying genes with high expression. The Snapgene design program is used to clone the vaccine construct into plasmid pET-28a (+) (56) (**Figure 1**).

## 3 Results

### 3.1 Retrieval of EC- related viral sequences

Ten FASTA-formatted protein sequences from viruses related to esophageal cancer, with differing lengths of amino acids, were acquired from UniProt. Their antigenicity was checked and non-antigenic proteins were removed. Five proteins were identified as allergenic and are being examined further. The details of these proteins are reported in (**Table 1**).

**Table 1:** Details of selected antigenic vaccine candidates.

Virus	Proteins	Accession No	Antigenicity
Human Papillomavirus (HPV)	E6 oncoprotein	P03126	0.6921
	E7 oncoprotein	P03129	0.5765
Epstein-Barr Virus (EBV)	EBNA1 (Epstein-Barr Nuclear Antigen 1)	P03211	0.5545
Human Immunodeficiency Virus (HIV)	Tat (Transactivator of transcription)	P04610	0.7411
	Nef (Negative regulatory factor).	P04324	0.6258

### 3.2 Epitopes prediction and evaluation phase

HTL, CTL, and B-cell epitopes for particular antigenic proteins were predicted by the study. For vaccine design, the top eight CTL and eight LBL epitopes were selected because they are non-toxic, immunogenic, antigenic, and allergy-free (**Tables 2**, **3**). Likewise, three HTL epitopes were chosen due to their IFN-γ-inducing, non-toxic, non-allergic, immunogenic, and antigenic qualities (**Table 4**).

**Table 2:** Top eight CTL Epitopes selected for construct designing.

Protein	Epitopes	Alleles	Position	Immunogenicity	Antigenicity	Allergenicity
Protein E6	FRDLCIVYR	HLA-C*07:01 HLA-B*27:05 HLA-C*08:0 HLA-C*04:01	54-62	0.10	2.1	Non-Allergen
	SRTRRETQL	HLA-B*14:02 HLA-C*07:02 HLA-C*06:02 HLA-B*27:05	150-158	0.17	0.9	Non-Allergen
Protein E7	RAHYNIVTF	HLA-A*32:01 HLA-B*15:01 HLA-B*35:01 HLA-B*57:01 HLA-B*46:01 HLA-A*23:01 HLA-B*48:01 HLA-A*24:02 HLA-C*03:03	49-57	0.18	0.5	Non-Allergen
EBNA1	DLPPWFPPM	HLA-E*01:01 HLA-A*26:01 HLA-C*07:02	605-613	0.29	0.7	Non-Allergen
TAT	KRRQRRRPP	HLA-B*14:02 HLA-C*07:02 HLA-A*30:01 HLA-C*06:02	51-59	0.03	0.5	Non-Allergen
NEF	RAEPAADGV	HLA-C*15:02 HLA-C*05:01 HLA-C*12:03	22-30	0.13	0.8	Non-Allergen
	YPLTFGWCY	HLA-B*35:01 HLA-B*53:01 HLA-A*29:02 HLA-B*18:01 HLA-B*51:01	135-143	0.33	1.3	Non-Allergen
	VARELHPEY	HLA-B*35:01 HLA-B*46:01 HLA-A*29:02	194-202	0.18	1.1	Non-Allergen

**Table 3:** Eight LBL epitopes selected for the designing of a multi-epitope vaccine.

Protein	Epitopes	Position	Immunogenicity	Allergenicity	Antigenicity
Protein E6	RDLCIVYRDGNPYAVC	55	0.2	Non-Allergen	1.0
Protein E7	DRAHYNIVTFCCKCDS	48	0.2	Non-Allergen	0.6
	STHVDIRTLEDLLMGT	71	0.2	Non-Allergen	0.7
EBNA1	HGRGRGRGRGRGGGRP	39	0.4	Non-Allergen	1.2
	RGRGRGRGEKRPRSPS	370	0.04	Non-Allergen	1.2
NEF	EEKVGFPVTPQVPLRP	63	0.1	Non-Allergen	1.0
	GPGIRYPLTFGWCYKL	130	0.3	Non-Allergen	0.7
	EWRFDSRLAFHHVARE	182	0.3	Non-Allergen	0.9

**Table 4:** Final HTL Epitopes of EC proteins selected for construct designing.

Protein	Epitopes	Alleles	Antigenicity	Position	Immunogenicity	IFN-inducer
Protein E7	DLLMGTLGIVCPICS	HLA-DRB1*11:04, HLA-DRB1*11:06, HLA-DRB1*13:11	1.0	81-95	0.10	Positive
EBNA1	AGAGGAGAGGAGAGG	HLA-DQA1*05:01/DQB1*03:01	0.6	93-107	0.37	Positive
NEF	EVLEWRFDSRLAFHH	HLA-DRB1*03:05, HLA-DRB3*01:01, HLA-DRB1*03:06, HLA-DRB1*11:28, HLA-DRB1*13:05, HLA-DRB1*15:02, HLA-DRB1*11:07	1.0	179-193	0.53	Positive

### 3.3 Epitopes population coverage

**Figure 2:**
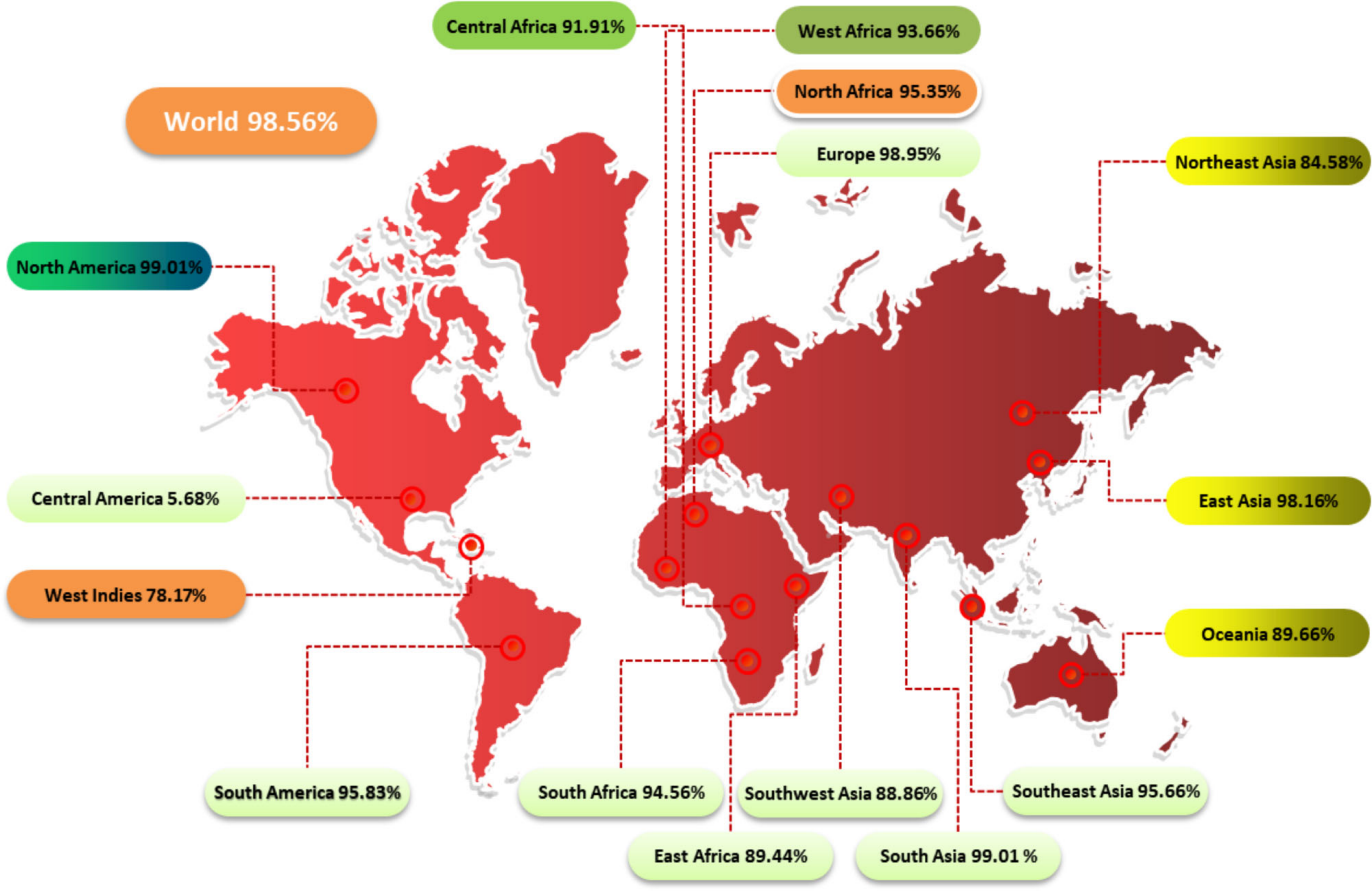
Population coverage graph of the designed vaccine construct.

The study examined the impact of PC on CD4+ and CD8+ T cells in 16 global regions. Results showed that North America and South Asia had the highest coverage of PC, while Central America received the least coverage at 5.68% (**Figure 2**). This information can help assess the efficacy of a vaccine against HLA alleles in different ethnic groups.

### 3.4 Vaccine construction

**Figure 3:**
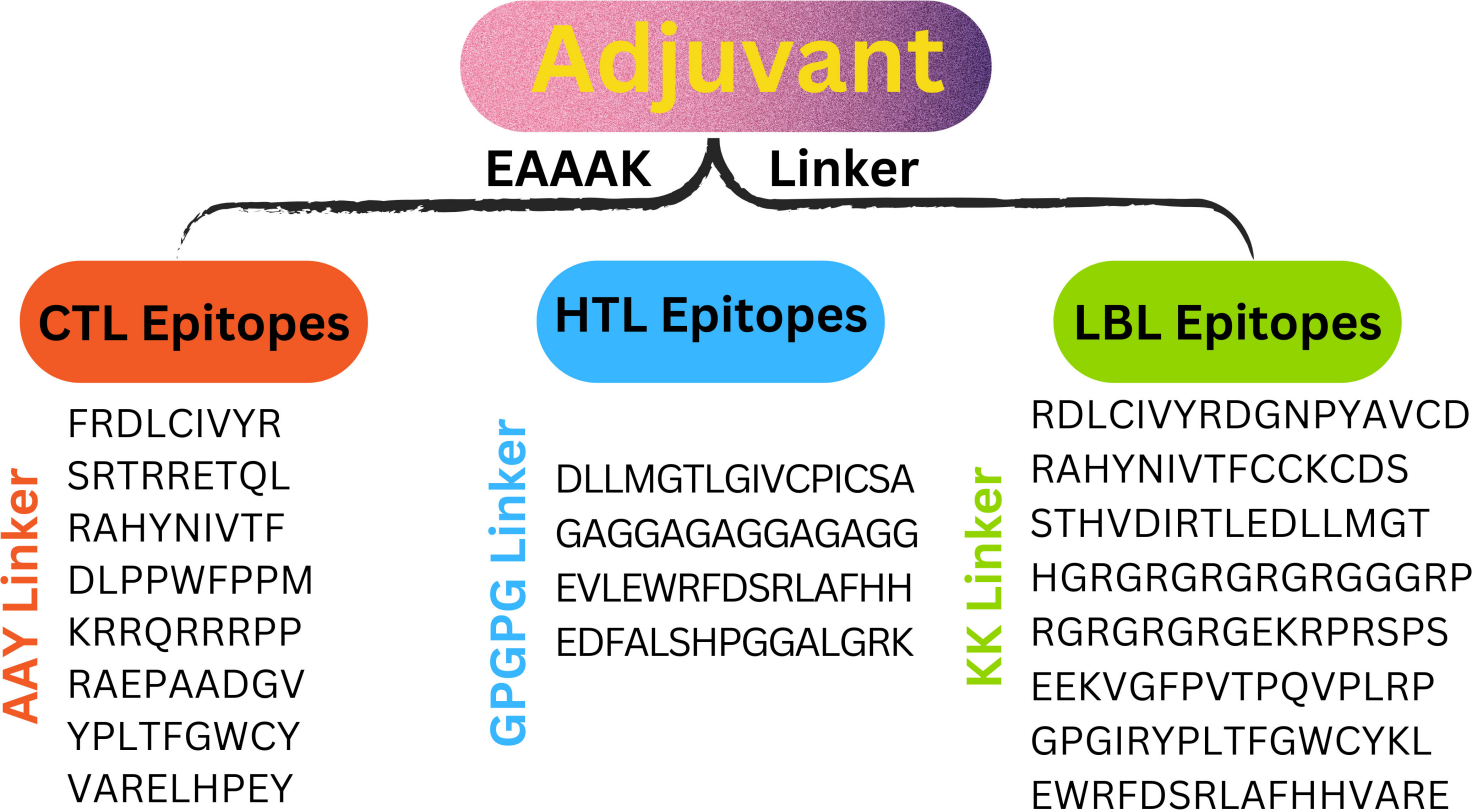
The designed vaccine construct is shown schematically.

Eleven T cells and eight linear B cell epitopes, each highly antigenic, non-toxic, and free of allergens, are part of the multi-epitope vaccine construct. Linkers from AAY, GPGPG, and KK have joined them. To attach the adjuvant β-defensin to the vaccine’s N-terminus, the EAAAK linker is utilized (**Figure 3** **).**

### 3.5 Post analysis of vaccine

#### 3.5.1 Prediction of physiochemical properties

The ProtParam server determined the final vaccine design’s MW to be 38 kDa and its amino acid composition to be 347. Our final construct is a suitable vaccine because its molecular weight is less than 110 kDa. There were 105 (Arg+Lys) positively charged residues in the vaccine. With an emphasis on yeast, mammalian reticulocytes, and E. coli, the study examined the lifespan of a multi-epitope vaccination. With a chemical formula of C1713H2704N536O453S19, the final vaccine was discovered to be highly soluble because of its polar nature and efficient water-binding interaction, as indicated by its -0.665 GRAVY and 59.14 aliphatic index. The anticipated structure had a 0.687530 probability of being soluble. Also evaluated were the vaccine’s non-toxicity, non-allergenicity, and non-antigenicity. VaxiJen predicted the final construct’s antigenicity to be 0.8621% at a virus model threshold of 0.5%. To make sure the potential vaccine did not result in toxic side effects or allergic reactions once it entered the body, its toxicity and allergenicity were evaluated and results revealed that the vaccine candidate was neither toxic nor allergic.

#### 3.5.2 Secondary & tertiary structure prediction

**Figure 4:**
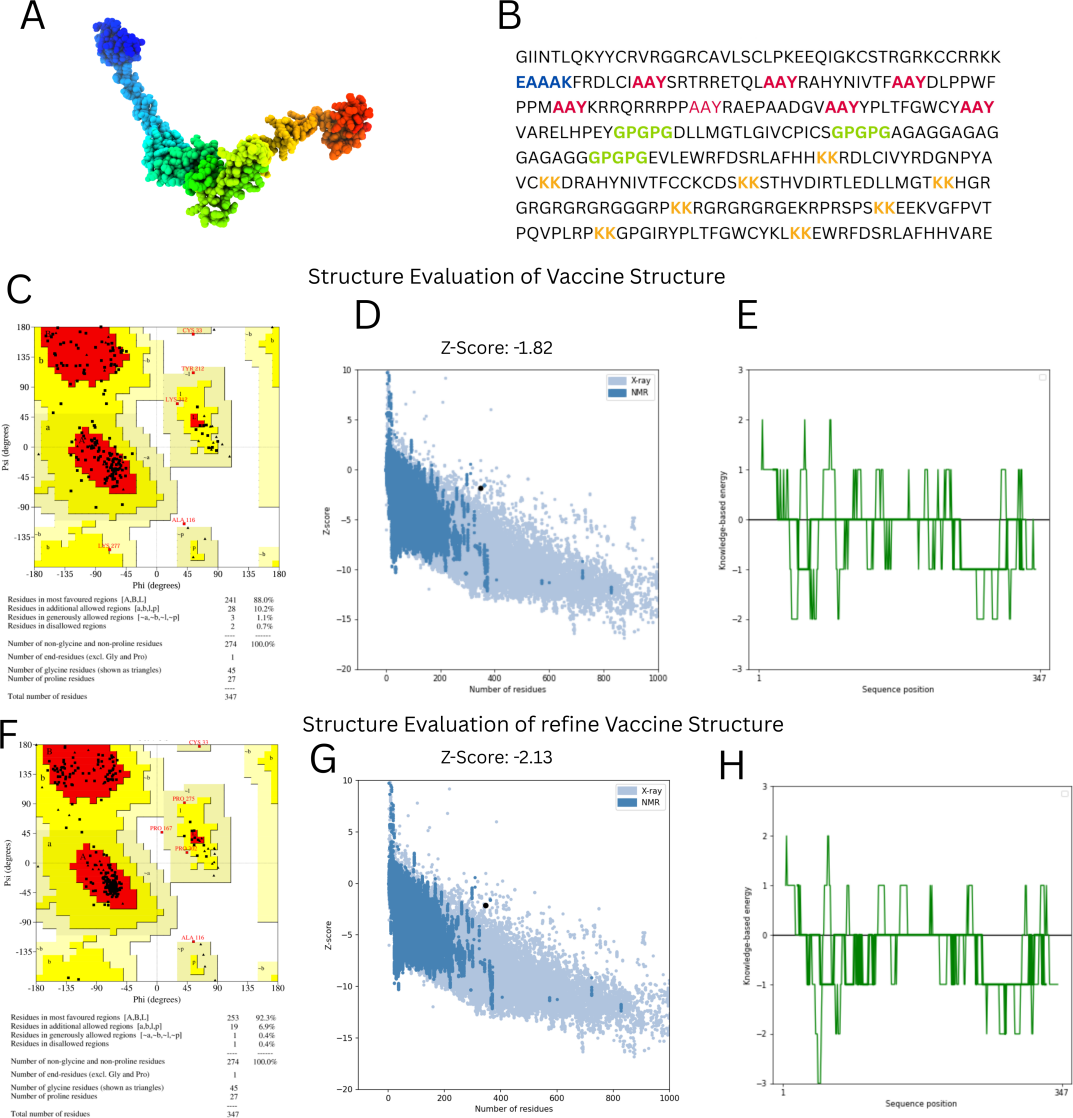
Structural modeling and validation of the multi-epitope vaccine construct **(A)** Tertiary structure of vaccine predicted using 3dpro **(B)** Vaccine sequence with adjuvant and linkers highlighted **(C, F)** Ramachandran plots before and after refinement respectively **(D, G)** Prosa-web Z-score plots showing model quality before and after refinement **(E, H)** ERRAT quality factor plots before and after refinement, conforming improved structural reliability.

According to the SOPMA server, the vaccine design is composed of 54.2% random coils, 23.3% β-strands, and 22.5% α-helices, showing a flexible yet organized conformation (**Figure 4A**). The final structure contains 347 amino acids.

Tertiary structure prediction was conducted with 3DPro and refined with GalaxyRefine (**Figure 4B**). Structural validation using the Ramachandran plot demonstrated improvement after refinement, with residues in favored areas increasing from 88.0% to 92.3% and disallowed regions falling from 0.7% to 0.4% (**Figures 4C, F**). The ProSA-web Z-score improved from -1.82 to -2.13, indicating higher model quality (**Figures 4D, G**). ERRAT analysis also indicated improved structural integrity after refinement (**Figures 4E, H**). These findings suggest that the vaccine structure is well-modeled and stable, making it appropriate for further interaction analysis.

#### 3.5.3 Vaccine B cell epitope prediction

**Figure 5:**
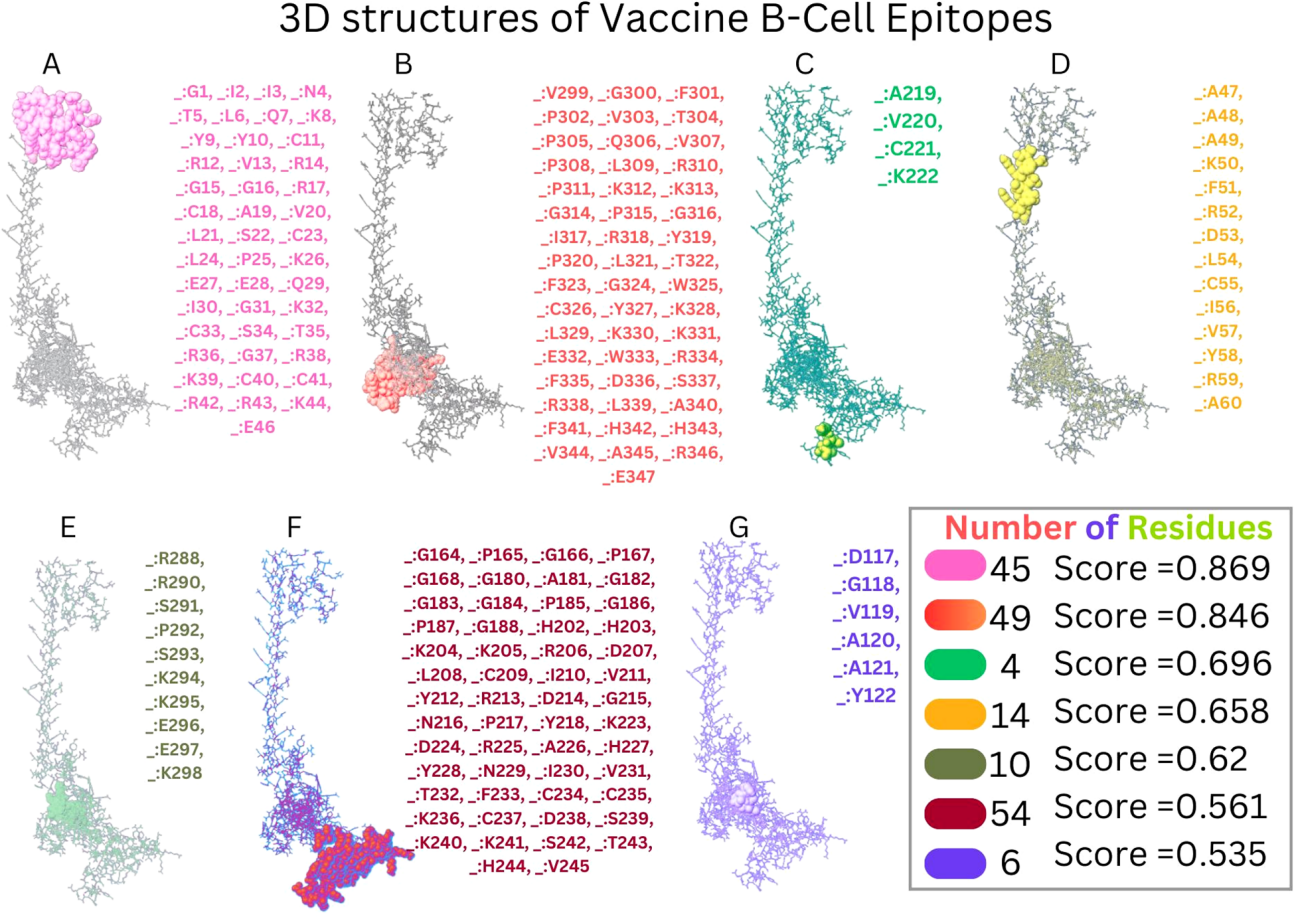
Conformational B cell epitopes of vaccine. **(A-G)** Presents the different B-Cell Epitopes with different colours.

With the use of the ElliPro server, B-cell epitopes inside the vaccine design were predicted, and five linear (continuous) epitopes, as detailed in **Table 5**, and eight conformational (discontinuous) epitopes, illustrated in **Figure 5**.

**Table 5:** Linear B cell epitopes of vaccine.

No.	Start	End	Peptide	Number of residues	Score
1	1	62	GIINTLQKYYCRVRGGRCAVLSCLPKEEQIGKCSTRGRKCCRRKKEAAAKFRDLCIVYRAAY	62	0.81
2	287	347	KRPRSPSKKEEKVGFPVTPQVPLRPKKGPGIRYPLTFGWCYKLKKEWRFDSRLAFHHVARE	61	0.797
3	202	245	HHKKRDLCIVYRDGNPYAVCKKDRAHYNIVTFCCKCDSKKSTHV	44	0.595
4	181	187	AGGGPGP	7	0.536
5	118	123	GVAAYY	6	0.529

### 3.6 Disulfide engineering

**Figure 6:**
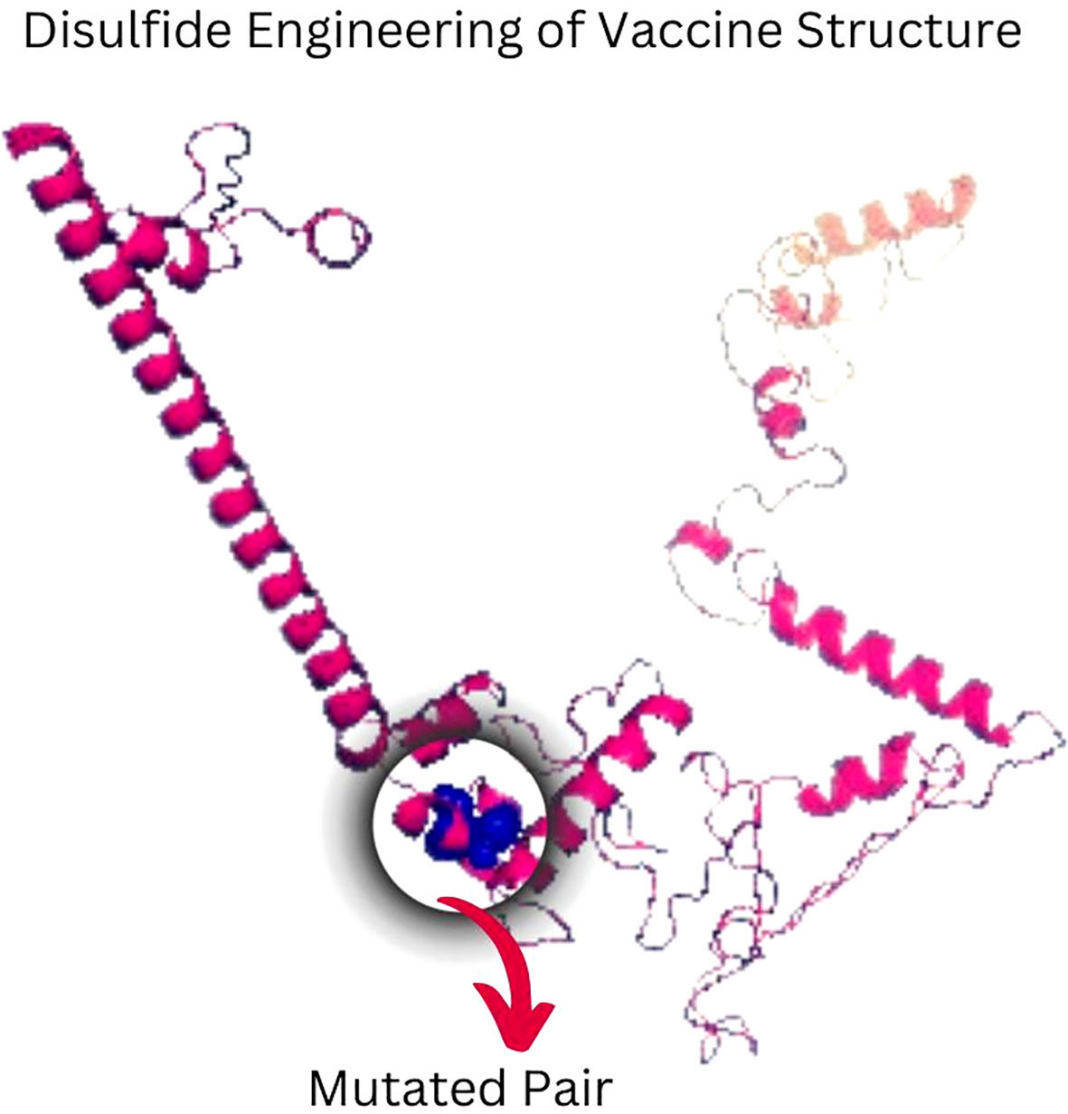
Disulfide engineering of vaccine construct improves stability. A mutant pair selected based on X3 value and energy is shown by the color blue.

The Disulfide by Design version 2.13 server was used to analyze the vaccine sequence, and it revealed twenty residue pairings that might potentially create disulfide bonds. Based on the energy of bonds and X3 characteristics, a pair of residues (107PRO-112ALA) was chosen since their results satisfied the requirements (**Figure 6**).

### 3.7 Immune simulation

**Figure 7:**
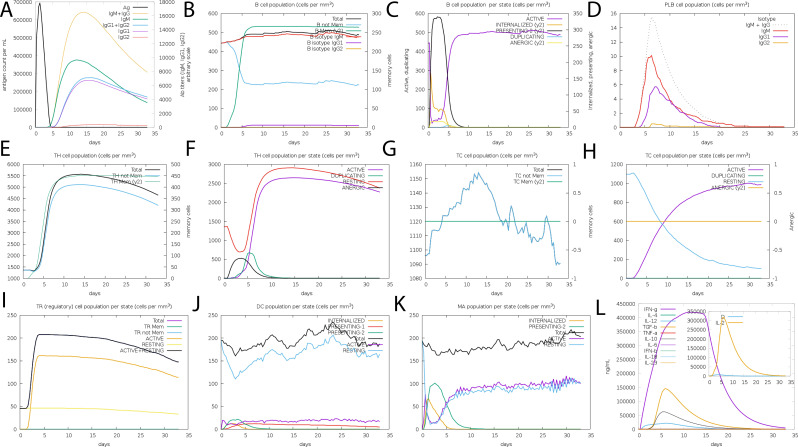
The vaccine’s immune profile. **(A)** Immunoglobulin concentrations in relation to antigens **(B)** Population of B-cell **(C)** Population of B-cell per state **(D)** Population of plasma B-cell **(E)** helper T-cell population **(F)** Population of helper T-cell per state **(G)** Cytotoxic T-cell population **(H)** Population of cytotoxic T-cell per state **(I)** T-regulatory cells reduced levels **(J)** Population of dendritic per state **(K)** Population of macrophage per state (**L**) The Simpson index of cytokine and interleukin production.

The *in silico* immune simulation projected an active B- and T-cell response to the suggested vaccine design, which is consistent with known immunological patterns. Initial high IgM levels indicated an initial immune response, followed by higher secondary and tertiary responses. The simulation also revealed the likelihood of memory B-cell growth and higher amounts of activated B cells, implying a long-term immune response. Immunoglobulin activity, comprising IgM, IgG1+IgG2, and combination IgG+IgM, was projected to remain consistently high, while the simulated vaccine concentration decreased over time (**Figures 7A–D**). The simulation also suggested a rise in cytotoxic T lymphocytes (CTLs), helper T lymphocytes (HTLs), and the development of memory Th and Tc cells (**Figures 7E–H** Regulatory T cells, dendritic cells, and macrophage populations were also expected to increase following simulated vaccine exposure. The simulation output showed higher amounts of cytokines, including IFN-γ and IL-2 (**Figures 7I–L**).

While these data show that the vaccine architecture may generate a strong immune response, it is important to note that they are computational predictions. Experimental validation is needed to confirm the immunogenicity and efficacy of the proposed formulation in biological systems.

### 3.8 Molecular docking

A vaccine’s immunogenic potential is greatly influenced by its affinity for immunological receptors such as toll-like receptors (TLRs), which play an important role in triggering host immune responses. In this investigation, the HADDOCK version 2.4 server was used to investigate the interaction between the created vaccine design and human TLR3. The docking simulation produced a HADDOCK score of -74.6 ± 3.8 kcal/mol, indicating good binding between the two molecules (**Table 6**).

**Table 6:** Docking statistics of TLR3-vaccine complex.

HADDOCK score	74.6 +/- 3.8
Cluster size	24
RMSD from the overall lowest-energy structure	45.2 +/- 0.3
Van der Waals energy	-55.0 +/- 4.4
Electrostatic energy	-250.7 +/- 60.5
Desolvation energy	-16.8 +/- 5.2
Restraints violation energy	1965.5 +/- 100.9
Buried Surface Area	2317.0 +/- 159.4
Z-Score	-1.3

**Figure 8:**
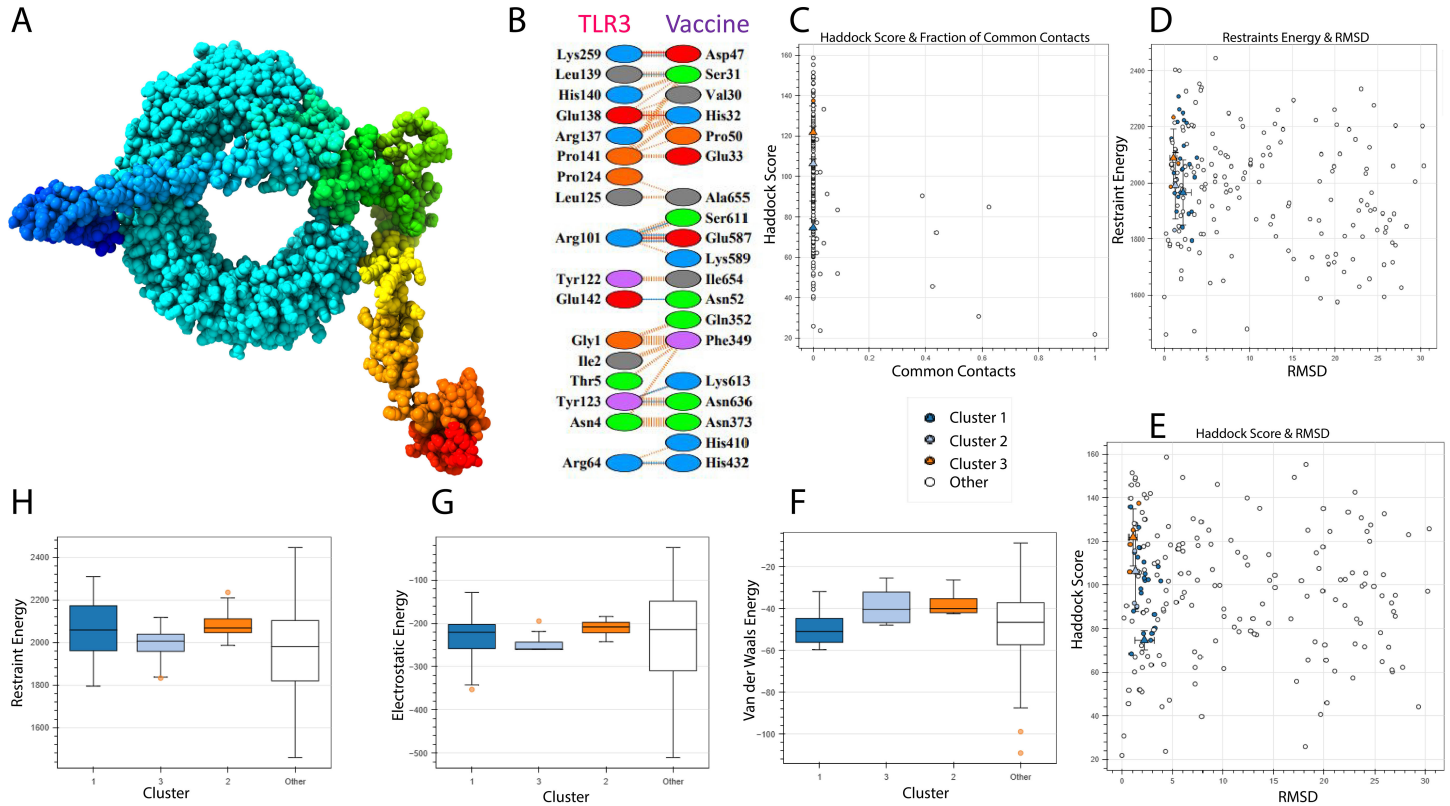
TLR-3 receptor and vaccine docked. TLR3 is shown in blue, while the vaccine is shown in rainbow colors. **(A)** 3D Structure visualization **(B)** Interaction analysis between vaccine and TLR3 **(C)** Docking score and common contacts **(D)** Energy calculations and RMSD **(E)** Haddock score and RMSD **(F, G)** Energy analysis including electrostatic, van deer waals.

**Figure 8A** depicts the docked complex, with TLR3 in blue and the vaccine construct in rainbow colors. PDBsum analysis revealed nine hydrogen bonds with an average bond length of around 3.34 Å, indicating a stable contact interface (**Figure 8B**).

Although the docking analysis showed a rather high root-mean-square deviation (RMSD) of 45.2 Å, this can be attributed to the flexible and complex structure of the multi-epitope vaccine, which may result in a larger range of anticipated conformations (**Figure 8C–H**). Despite this, the vaccine’s high HADDOCK binding score and the existence of many hydrogen bonds indicate a persistent and relevant interaction with TLR3. However, based on the combined docking score and interaction analysis, the results are deemed reliable within the context of this computational investigation and indicate the vaccine’s ability to engage immune receptors.

### 3.9 MD simulation

**Figure 9:**
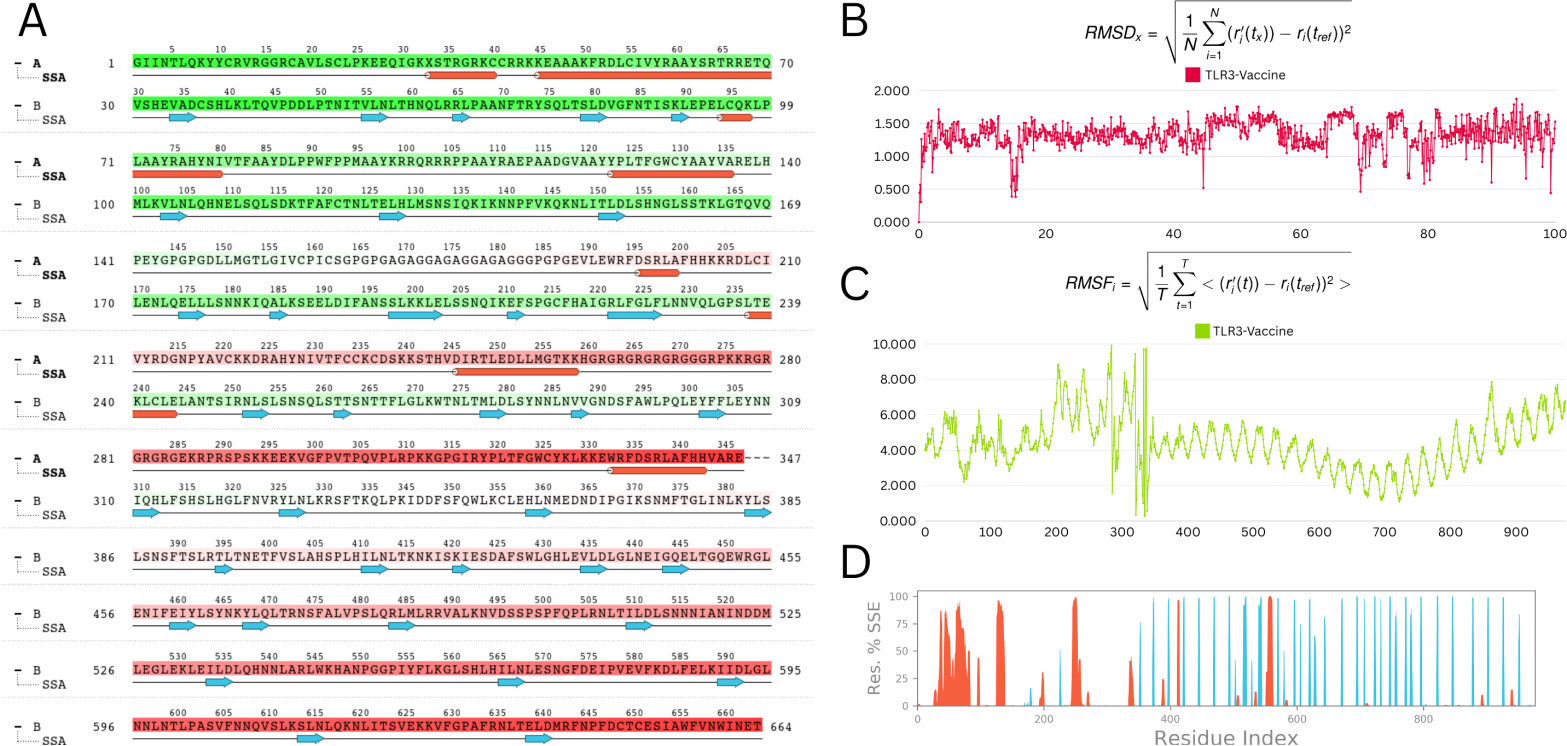
Molecular dynamic simulation analysis **(A)** Secondary structure analysis (SSA): This panel illustrates the dynamic changes in the secondary structure elements (α-helices, β-strands, and loops) of the protein throughout the simulation time. The color-coded representation allows for an easy visual interpretation of the stability and transitions between different secondary structures **(B)** Root mean square deviation (RMSD): The RMSD plot depicts the deviation of the protein’s backbone atoms over the course of the simulation. The RMSD values are stable, fluctuating within a narrow range of 1 to 1.5 Å, indicating that the protein structure remains stable throughout the simulation **(C)** Root mean square fluctuation (RMSF): The RMSF plot shows the flexibility of individual residues during the simulation. Most residues exhibit minor fluctuations, with the majority maintaining stability. Notable minor fluctuations are observed between residues 250 to 300, which may correspond to flexible regions or loop areas of the protein **(D)** Residue index vs. secondary structure elements (SSE): This panel provides a detailed view of the secondary structure assignment for each residue throughout the simulation. It highlights the regions of the protein that maintain their secondary structure or undergo transitions, offering insights into the dynamic behavior of specific segments.

A 100-nanosecond molecular dynamics (MD) simulation was performed to determine the vaccine-TLR3 complex’s structural stability and flexibility. The root-mean-square deviation (RMSD) analysis revealed initial oscillations at 10.9 Å around 20 ns, followed by overall stabilization with an average RMSD of 4.0 Å, indicating a relatively stable complex throughout the simulation (**Figure 9B**).

Root-mean-square fluctuation (RMSF) study showed minimal residue-level flexibility, with an average variation of 2.9 Å and a maximum of 3.6 Å. TLR3 had consistent behaviour in the presence of the vaccine, indicating minimal disruption upon binding (**Figure 9C**).

Analysis of the radius of gyration (Rg) verified the structural compactness over time. The structural robustness of important protein areas was demonstrated by secondary structure analysis (SSA), which revealed persisting α-helices and β-strands throughout the simulation (**Figures 9A, D**). The vaccine–receptor complex’s tight interface and dynamic stability are often supported by MD studies. Overall, MD results are consistent with the vaccine-receptor complex’s dynamic stability and compact interaction.

### 3.10 *In-silico* cloning

**Figure 10:**
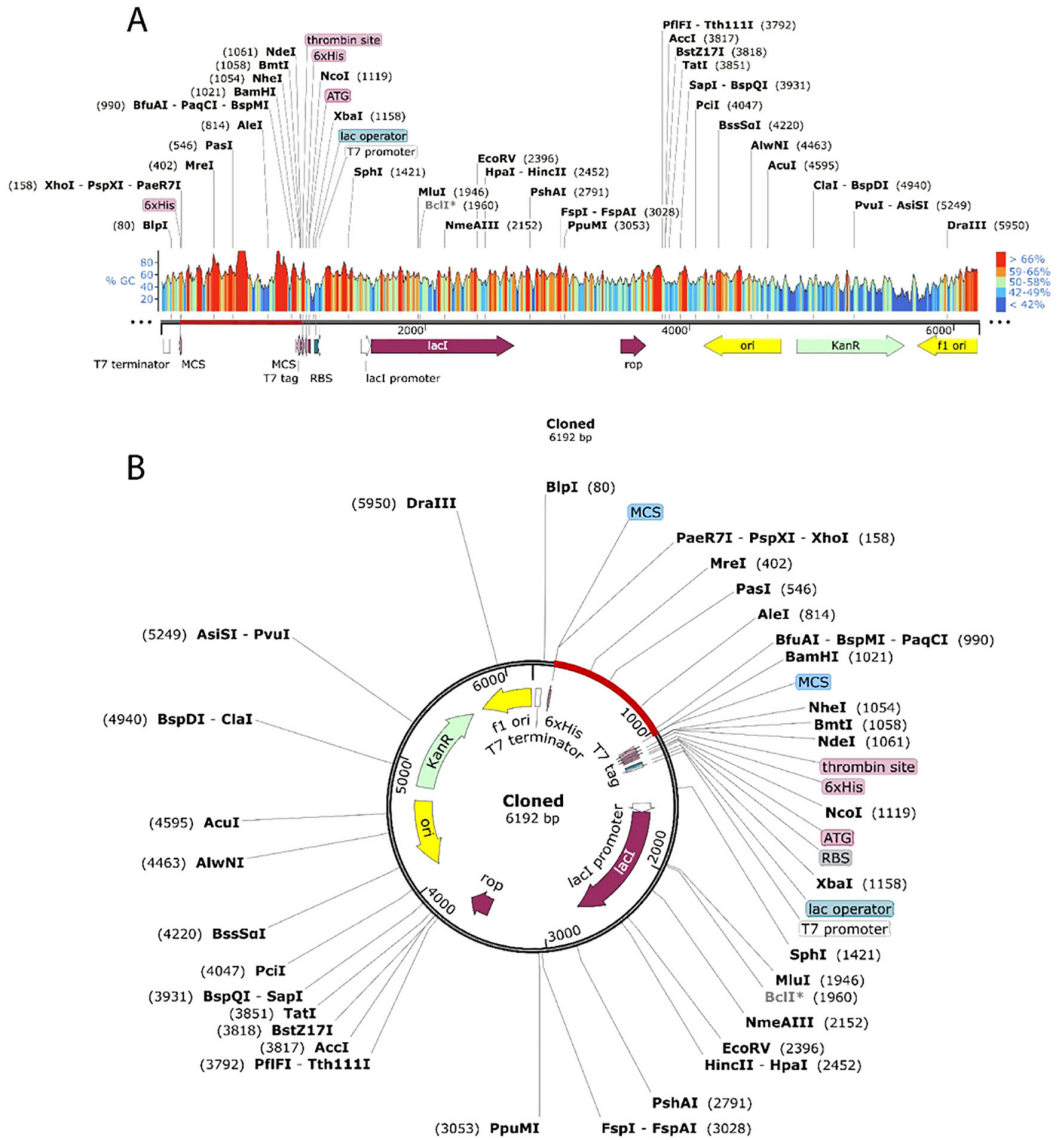
Cloning the vaccine *in silico* using the pET28a (+) expression vector. Cloned region is highlighted by red color.

The JCat tool was used to optimize the vaccine’s codon utilization for the E. coli K12 strain, yielding a 55.3% GC content and 1 CAI, signifying successful expression in the E. coli host. Although a GC percentage somewhat higher than 50% might sometimes influence expression efficiency, it is still within an acceptable range, and codon harmonization helps avoid such concerns by matching codon usage with the host’s tRNA availability. The pET28a(+) E. coli expression vector was altered by incorporating the codon sequence between the XhoI and BamHI restriction sites. (**Figure 10A**), resulting in a recombinant construct of 6192 base pairs (**Figure 10B**) appropriate for future experimental validation and protein expression analysis.

## 4 Discussion

Esophageal cancer’s aggressive progression and late-stage diagnosis make it difficult to treat, highlighting the need for better treatment plans and early detection. A multi-epitope vaccine could target multiple antigens on cancer cells, enhancing the immune system’s ability to combat diverse tumors, reducing immune evasion and tumor recurrence, and potentially providing a more potent and individualized therapeutic approach (12, 57–60).

Vaccines based on peptides, especially those that contain several epitopes, have shown great promise in producing immune responses against a variety of malignancies, including esophageal cancer (EC). Numerous multi-epitope cancer vaccines have advanced through Phase I and II clinical studies, demonstrating positive immunogenicity, safety profiles, and tolerability (61–64). Examples include HER2-derived peptides (E75, AE37, and GP2) in breast cancer (65–67) and gp100 or MART-1 peptides in melanoma, which have induced antigen-specific T-cell responses in early-phase investigations (68, 69). Although several Phase III trials, such as nelipepimut-S with GM-CSF in breast cancer, did not show substantial increases in survival outcomes, these findings highlight the complexities of tumour immunology rather than the ineffectiveness of peptide vaccines themselves (70). Recent developments, such as the application of contemporary adjuvants like poly-ICLC, have significantly improved T-cell activation in early-phase glioblastoma trials, underscoring the field’s continuous advancement (71). The continuous development of peptide vaccines is supported by the fact that they are still well tolerated and rarely cause serious side effects.

It is critical to understand that viral oncogenesis in esophageal cancer (EC) can entail ‘hit-and-run’ mechanisms, in which viral proteins trigger malignant transformation but are no longer produced in advanced tumours. This is a hurdle for therapeutic vaccines targeting viral antigens in late-stage disease. Hence, our multi-epitope vaccine is primarily intended as a preventive method, stimulating immune responses against viral components before malignant transformation starts. Conceptually, this strategy is consistent with effective preventative vaccines, like those that target HPV. Our vaccine, which incorporates multiple virus-associated epitopes relevant to EC, aims to provide broad, protective immune activation and is a candidate for future preclinical and clinical development in virus-linked cancer prevention, given the encouraging outcomes of peptide-based vaccines in early-phase cancer trials and their excellent safety profiles.Ten proteins from various viruses were chosen to predict effective epitopes in light of the significance of the virulence factors. The protein targets chosen for a broad-spectrum therapeutic vaccine targeting virus-associated cancers include E6, E7, LMP1, EBNA1, IE proteins, UL97, LANA, vIL-6, Tat, and Nef. These proteins are critical in viral replication, immune evasion, and oncogenesis, and are consistently expressed in infected or transformed cells, making them promising candidates for designing a vaccine. Their prior application in studies on vaccines or immunotherapy, supports their inclusion as potential protective antigens (72–76). Non-antigenic proteins were eliminated once their antigenicity was examined. Five antigenic proteins were taken into consideration for additional examination. HTL, CTL, and LBL epitopes were used to identify the target proteins, and the multi-epitope candidate was selected based on the antigenicity, toxicity, immunogenicity, and allergenicity of the proteins. Linkers can enhance rigidity, folding, and expression in vaccine development. The vaccine was constructed by joining the CTL, LBL, and HTL epitopes with AAY, KK, and GPGPG linkers, respectively, as other previous studies have demonstrated (77–81). Since flexible, hydrophilic amino acids normally make up GPGPG, AAY, and KK linkers, combining these two residues can stop folding disruption and domain function (77, 82). β-defensin was used as an adjuvant in the EAAAK linker. Owing to its immunomodulatory and antibacterial qualities, β-defensin is a useful adjuvant that has been employed in numerous prior investigations (83–86). Studies in the past have demonstrated that this a-helical linker’s inflexible structure gives protein domains adequate room to fold and perform independently. Furthermore, stability—particularly thermal stability, which is crucial for vaccines—can be significantly increased by incorporating this linker into the fusion proteins. The final vaccine was 347 amino acids long, but some studies have found much larger vaccine sizes (77, 81, 87–89). As a result, we believe it will not interfere with consistency or interpretation.

To ensure a vaccine’s efficacy, it must provide broad-spectrum immunity across diverse global populations. Therefore, understanding the HLA genotypic frequencies within the target endemic regions is essential for vaccine development. According to the statistics, the chosen CTL and HTL epitopes represent a considerable fraction of the global population.

The designed vaccine’s appropriate molecular weight facilitates purification, making it a suitable vaccine. Greater thermostability at various temperatures is indicated by high aliphatic index values, while negative GRAVY values reflect the candidate vaccine’s hydrophilic nature, which allows it to form strong bonds with water molecules. Furthermore, the vaccine has good solubility.

Recognizing viral particles and activating the innate immune system depend on TLRs. TLR targeting may be crucial for creating vaccines and averting illness (90, 91). Molecular docking was used to examine the vaccine’s specific interactions and binding affinities against TLR3. A strong binding affinity was shown by the energy scores attained when binding the vaccine-TLR3 complex. In conclusion, the study’s MD simulation results validate that the vaccine molecule can engage in optimal interaction with the TLR3 protein.

The proposed vaccine’s ability to elicit an immune response was then evaluated utilizing an immunological simulation and the C-IMMSIM server. However, memory T and B cells appeared to have significantly improved, according to the data. There was also a noticeable increase in the IFN-γ titer and a slight rise in IL-2 following the third vaccination shot, while in the primary immune response, antibody levels were lower than in the tertiary and secondary immune responses. Together, these findings imply our potential multi-epitope vaccine may effectively elicit an immunological response, resulting in a robust resistance against infections.

To maximize the multi-epitope vaccine’s translation efficiency inside a particular expression system, the vaccine’s mRNA was then amplified using JCAT. The expression vector pET28a (+) was constructed by incorporating adaptive DNA sequences at both the C- and N-termini, between the BamHI and XhoI restriction sites. A CAI value of 0.98 and a GC content of 53.63% in bacteria suggested high-level protein expression.

While the preliminary results appear encouraging, it is important to recognize several limitations. The initial vaccine design, based on computer projections and simulations, may not fully capture *in vivo* responses. While computational predictions like epitope immunogenicity, antigenicity, and population coverage offer valuable insights, these must be validated through experimental approaches like ELISpot assays, flow cytometry, and *in vivo* studies using animal models. Prioritizing experimental validation in preclinical and clinical environments is crucial for ensuring the vaccine’s safety and efficacy. Numerous studies have shown that *in silico*-designed vaccine candidates have shown high efficacy in experimental evaluations (92–94). Hence, the outcomes of this study can serve as the solid framework for future exploration meant to precisely ascertain the vaccines’ therapeutic potential.

Our vaccine construct is envisioned primarily as a therapeutic vaccine aimed at eliciting robust cellular and humoral immune responses against viral epitopes expressed by esophageal cancer cells. While preventive vaccines target viral infection before tumor development, therapeutic vaccines focus on controlling or eradicating established malignancies. The immunoinformatics predictions provide a foundation for this approach, but further experimental validation is essential to confirm efficacy in clinical settings.

## 5 Conclusion

Globally, esophageal cancer is among the most prevalent cancers. The primary viruses involved in the pathophysiology of EC are HIV, HHV-8, HCMV, EBV, and HPV. To counter these important epitopes, an in silico vaccine was developed in this work, and its effectiveness was evaluated using a variety of immunoinformatics servers. The outcomes showed a rise in T cells, including cytotoxic and helper T cells, as well as an increase in INF-γ and antibodies, indicating that the multi-epitope vaccine that was designed serves as a useful prophylactic candidate vaccine. Further validation through *in vitro* and *in vivo* investigations is required to establish the vaccine’s immunogenicity and safety, allowing for its potential use in public health strategies.

## Data Availability

The datasets presented in this study can be found in online repositories. The names of the repository/repositories and accession number(s) can be found in the article/supplementary material.
